# Supplementary material for: Role of TMEM100 in mechanically insensitive nociceptor un-silencing
Source: Nat Commun. 2023 Apr 5;14:1899. doi: 10.1038/s41467-023-37602-w (PMC10076432; doi:10.1038/s41467-023-37602-w)
Supplement: Supplementary file 1 — Supplementary Information [file 41467_2023_37602_MOESM1_ESM.pdf]

Supplementary Information

Role of TMEM100 in mechanically insensitive nociceptor un-silencing.

Author list

Timo A. Nees[1,2], Na Wang[3], Pavel Adamek[4], Nadja Zeitzschel[4], Clement Verkest[4], Carmen La Porta[1], Irina Schaefer[1], Julie Virnich[4], Selin Balkaya[4], Vincenzo Prato[1], Chiara Morelli[5], Valerie Begay[6], Young Jae Lee[7], Anke Tappe-Theodor[1], Gary R. Lewin[6], Paul A. Heppenstall[5], Francisco J. Taberner[1,8], Stefan G. Lechner[1,4,*]

Affiliations

[1] Institute of Pharmacology, Heidelberg University, Im Neuenheimer Feld 366, 69120 Heidelberg, Germany

[2] Department for Orthopeadics , Heidelberg University Hospital, Schlierbacher Landstrasse 200a, 69118 Heidelberg, Germany

[3] Institute of Pathophysiology, Yan'an University, Shengdi Road 580, 716000 Yan'an, China

[4] Department of Anesthesiology, University Medical Center Hamburg-Eppendorf, Martinistrasse 52, 20246 Hamburg, Germany

[5] SISSA: Scuola Internazionale Superiore di Studi Avanzati, Via Bonomea, 265, 34136 Trieste, Italy.

[6] Department of Neuroscience, Max Delbrück Center for Molecular Medicine, Berlin, Germany.

[7] Department of Biochemistry, Lee Gil Ya Cancer and Diabetes Institute, Gachon University College of Medicine, Incheon, Republic of Korea

[8] Instituto de Neurosciencias de Alicante, Universidad Miguel Hernández – CSIC, Alicante, Spain

*Correspondence: s.lechner@uke.de

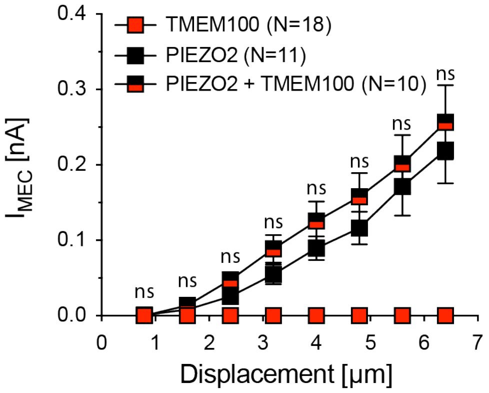

Supplementary Figure 1, TMEM100 transfected HEK293 cells do not acquire mechanosensitivity nor modulate PIEZO2 mediated mechanotransduction currents. To assess the mechanosensitivity of TMEM100 and of PIEZO2 in the presence (PIEZO2 + TMEM100) and absence of TMEM100 (PIEZO2), the constructs were (co)-transfected into HEK293 cells using the calcium phosphate method. Mechanosensitivity was assessed 48h after transfection using the mechano-clamp technique. Mean ± SEM peak amplitudes of mechanically-evoked currents are shown as a function of membrane displacement for TMEM100 (red square, N=18), PIEZO2 (black square, N=11) and PIEZO2+TMEM100 (black/red square, N=10) transfected HEK293 cells. When expressed in HEK293 cells, TMEM100 did not produce mechanotransduction currents, whereas PIEZO2 transfected cells acquire mechanosensitivity and show robust mechanotransduction currents, which are not modulated by co-expression of TMEM100. Since TMEM100-expressing cells did not produce mechanotransduction currents, a statistical comparison was only performed for PIEZO2 vs. PIEZO2+TMEM100 using multiple two-sided Mann-Whitney tests (P-values from left to right: 0.99, 0.23, 0.14, 0.17, 0.28, 0.38, 0.66, 0.86). Source data are provided as a Source Data file

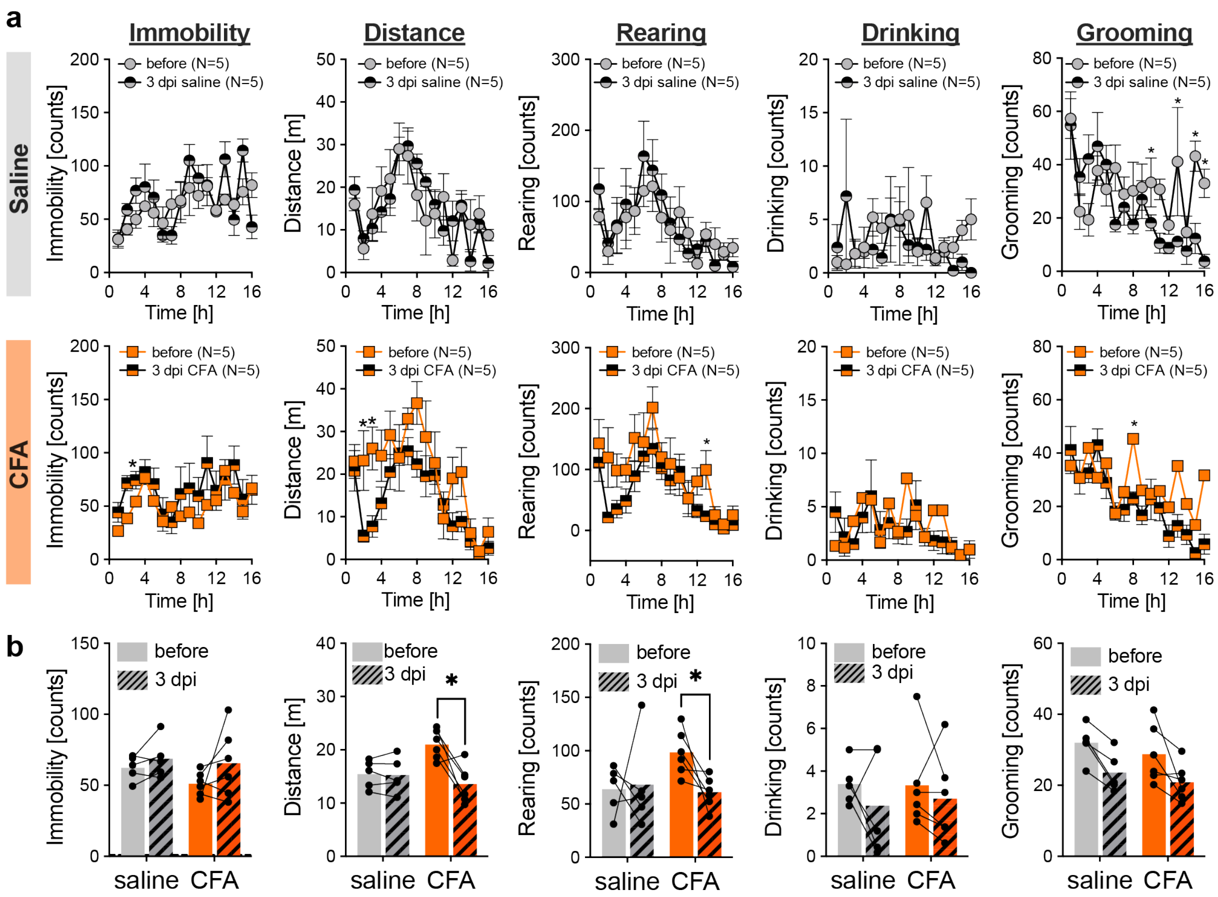

Supplementary Figure 2, Homecage behavior of WT mice following CFA-induced knee joint monoarthritis. Animals were analyzed continuously for 16-hours before and 3 days after saline (grey) or CFA (orange) injection. **a**, Data points represent the mean ± SEM counts per hour for immobility, rearing, drinking and grooming behavior and the mean ± SEM distance travelled per hour in meter (m). Homecage behavior was monitored over a time period of 16 hours (h). The upper panels display the behavior over time of the saline group before (grey circle) and 3 days after (dpi, grey/black circle) saline injection. The lower panels show the homecage behavior over time of the CFA group before (orange square) and 3 days after (orange/black square) CFA injection. Frequency counts per time point were compared using multiple two-sided Mann-Whitney tests (*, $P<0.05$). Exact P-values are provided alongside the raw data in the source data file. N-numbers are provided in the graph legends. **b**, comparison of the mean immobility, rearing, drinking and grooming counts as well as the mean distance travelled per test day before (solid bars) and three days after (3 dpi, hatched bars) saline (grey) and CFA (orange) injection. Two-sided paired Student's t-test (saline N=5, CFA N=5; *, $P^{distance,\ CFA} = 0.0176$, $P^{rearing,CFA} = 0.0345$). Source data are provided as a Source Data file.

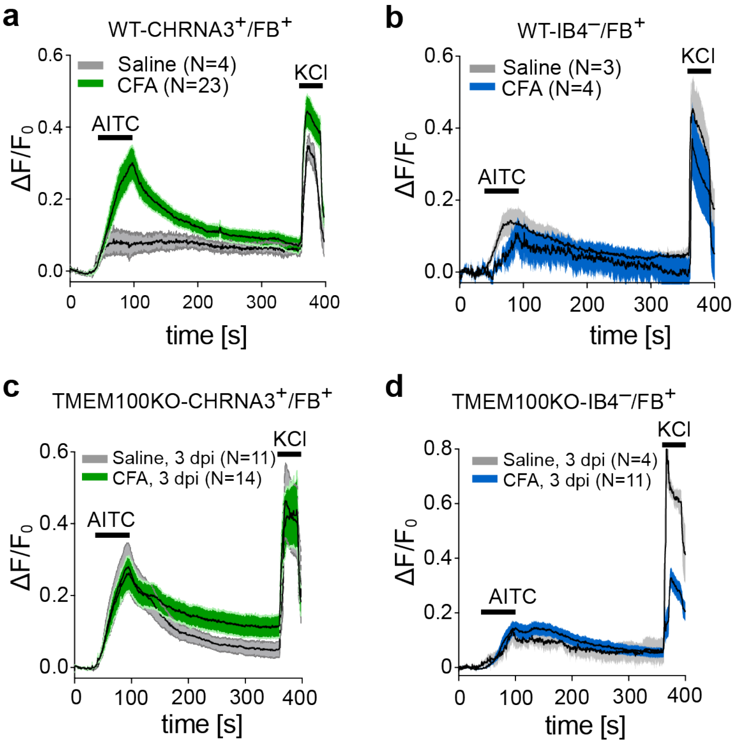

Supplementary Figure 3, TRPA1 activity in articular afferents.

a – **d**, time course of changes in the intracellular Ca^{2+} concentration ($\Delta F/F_0$) in response to 10 µM AITC (allyl isothiocyanate) and 100 mM KCl (potassium chloride) determined with Calbryte-590 Ca^{2+} imaging of retrogradely FB-labelled CHRNA3$^+$ articular MIAs (a and c) and putative polymodal articular peptidergic nociceptors (IB4$^-$/FB$^+$) (b and d) from saline and CFA treated wildtype (a and b) and TMEM100KO (c and d) mice. Lines show the mean responses ± SEM (shaded areas) from the indicated number of cells. Source data are provided as a Source Data file.

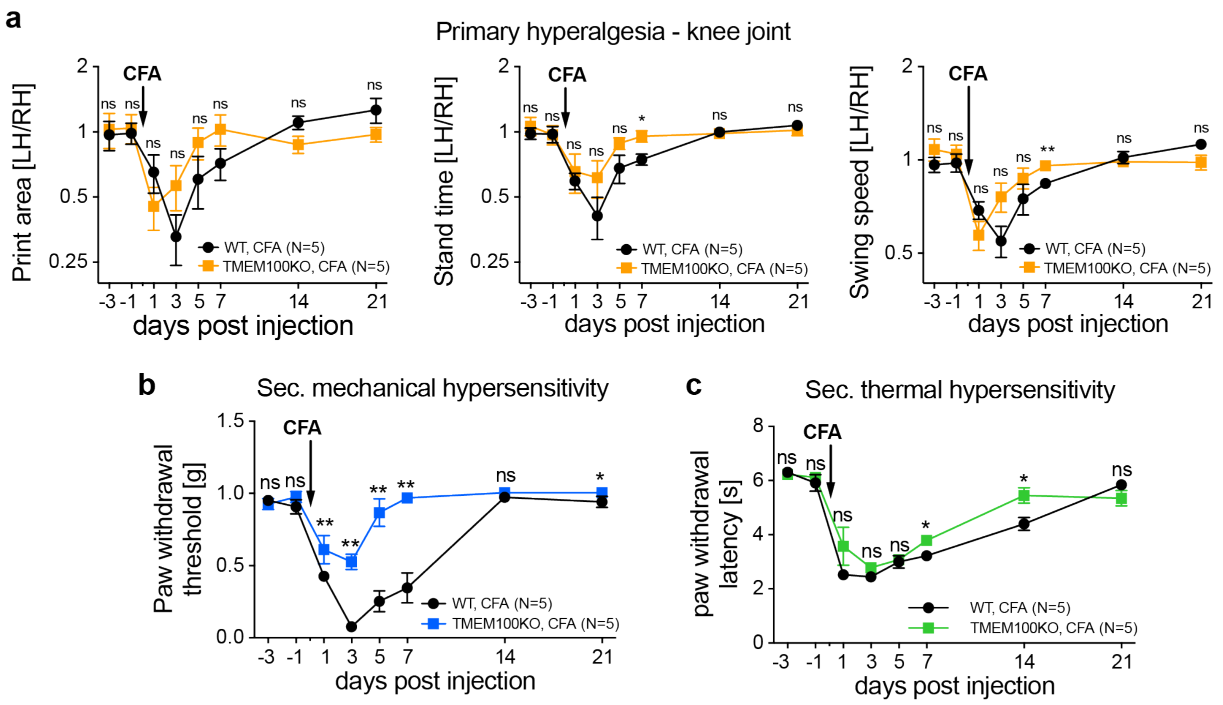

Supplementary Figure 4: Female TMEM100 knock-out mice develop normal inflammatory knee joint pain but no long-lasting secondary mechanical allodynia. a, Comparison of the time courses of changes in foot print area (left), stand time (middle) and leg swing speed (right) of CFA injected female WT mice (black circles) and CFA injected female TMEM100KO mice (orange squares). **b**, time courses of changes in mechanical paw withdrawal thresholds of CFA injected female WT mice (black circles) and CFA injected female TMEM100KO mice (blue squares). **c**, time courses of changes in thermal paw withdrawal latencies of CFA injected female WT mice (black circles) and CFA injected female TMEM100KO mice (green squares). **a – c** Symbols represent means ± SEM. Unless otherwise stated, ratios at different time points were compared using mixed model ANOVA. WT-CFA vs. TMEM100KO-CFA (ns, P > 0.05, *, P < 0.05; **, P < 0.01). Exact P-values are provided alongside the raw data in the source data file and N-numbers are provided in the graph legends. Source data are provided as a Source Data file.

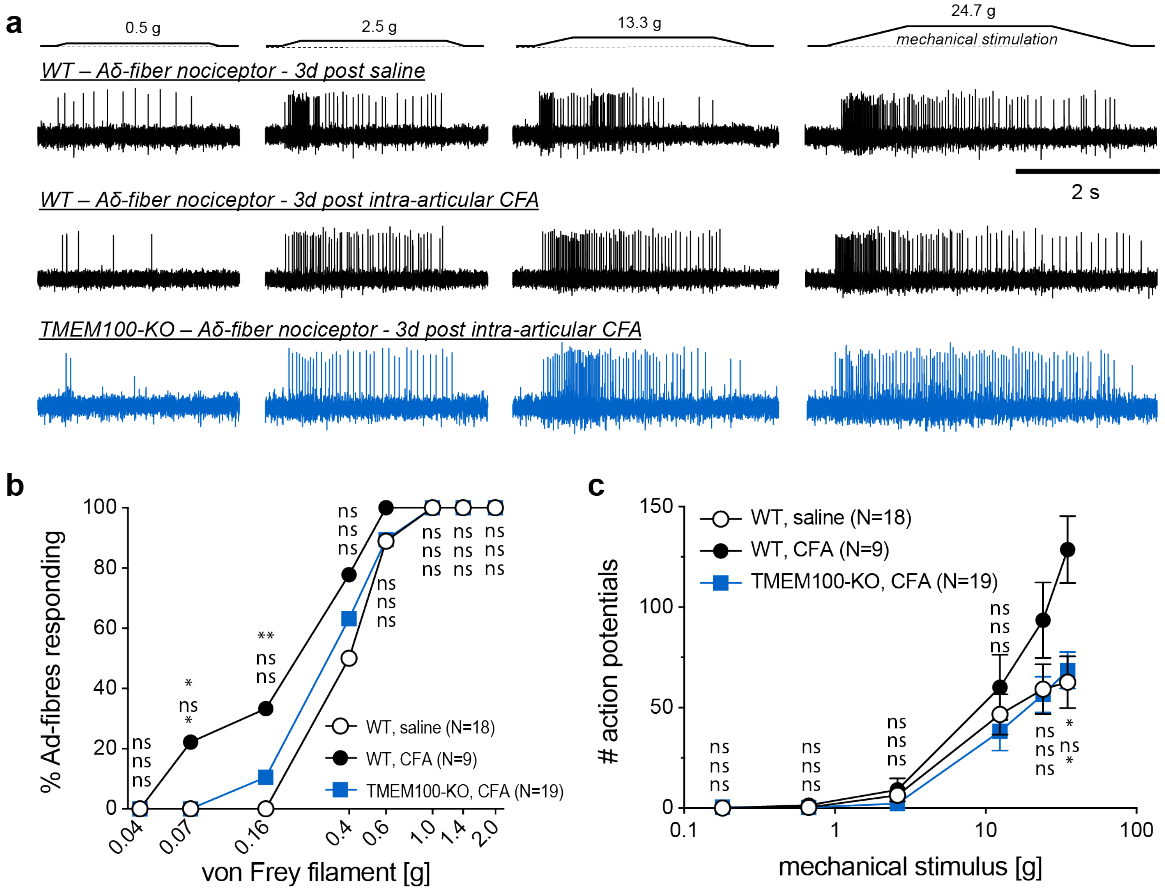

Supplementary Figure 5: Ex-vivo skin nerve recordings of cutaneous Aδ-fiber nociceptors following CFA-induced knee joint monoarthritis. a, Example traces of mechanically-evoked action potentials recorded from single nerve fibers from the tibial nerve of WT mice 3 days post intraarticular injection (dpi) of saline (top), WT mice 3 dpi of CFA (middle) and TMEM100KO mice 3 dpi of CFA (bottom). **b,** Comparison of the proportions of Aδ-fiber nociceptors that respond to mechanical stimulation with the indicated von Frey filaments. The proportions were compared pairwise using the two-sided Chi-square test. P-values are provided next to the symbols in the graph (ns, P>0.05; *, P<0.05; **, P<0.01) and refer to WT-saline vs. WT-CFA (top), WT-saline vs. TMEM100KO-CFA (middle) and WT-CFA vs. TMEM100KO (bottom). Exact P-values are provided alongside the raw data in the source data file and N-numbers are provided in the graph legend. **c,** Comparison of the firing rates evoked by a series of ramp-and-hold stimuli with increasing amplitudes that exerted the indicated force to the receptive fields. Symbols represent the mean ± SEM numbers of action potentials, which were compared using multiple two-sided Mann-Whitney tests. P-values are provided next to the symbols in the graph (ns, P>0.05; *, P<0.05) and refer to WT-saline vs. WT-CFA (top), WT-saline vs. TMEM100KO-CFA (middle) and WT-CFA vs. TMEM100KO (bottom). Exact P-values are provided alongside the raw data in the source data file and N-numbers are provided in the graph legend. Source data are provided as a Source Data file.

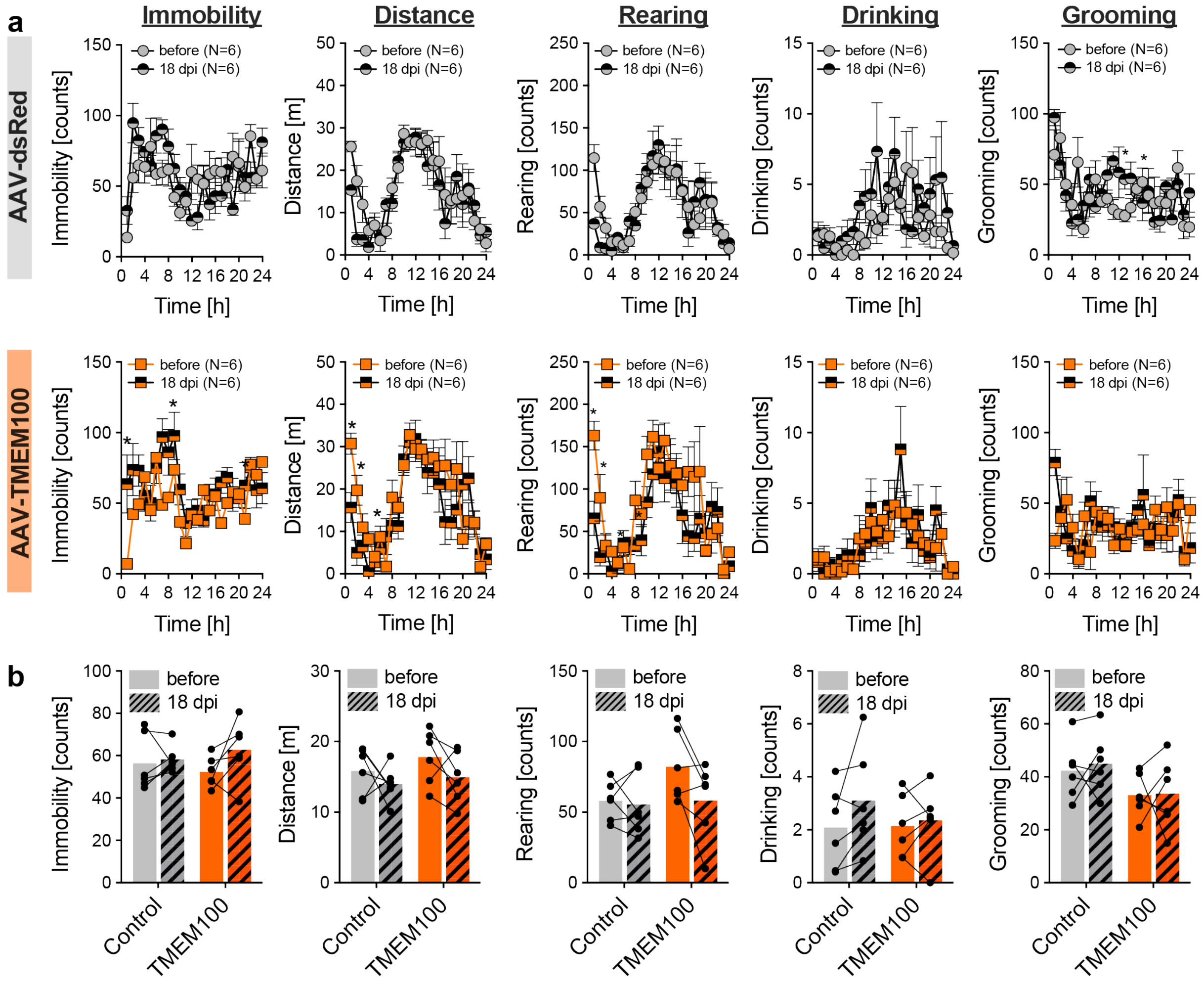

Supplementary Figure 6: Homecage behavior of WT mice following AAV-PHP.S-TMEM100-Ires-dsRed knee joint injection. Animals were analyzed continuously for 24-hours before and 18 days after intraarticular AAV-PHP.S-dsRed control virus (grey) or AAV-PHP.S-TMEM100-Ires-dsRed (orange) knee joint injection. **a**, Data points represent the mean ± SEM counts per hour for immobility, rearing, drinking and grooming behavior and the mean ± SEM distance travelled per hour in meter (m). Homecage behavior was monitored over a time period of 24 hours (h). The upper panels display the behavior over time of the control group before (grey circle) and 18 days after (dpi, grey/black circle) AAV-PHP.S-dsRed control virus injection. The lower panels show the homecage behavior over time of the CFA group before (orange square) and 18 days after (orange/black square) AAV-PHP.S-TMEM100-Ires-dsRed injection. Frequency counts per time point were compared using multiple two-sided Mann-Whitney tests (*, $P<0.05$). Exact P-values are provided alongside the raw data in the source data file and N-numbers are provided in the graph legends. **b**, comparison of the mean immobility, rearing, drinking and grooming counts as well as the mean distance travelled per test day before (solid bars) and 18 days after (3 dpi, hatched bars) AAV-PHP.S-dsRed control virus (grey) and AAV-PHP.S-TMEM100-Ires-dsRed (orange) injection. Two-sided paired Student's t-test (saline N=6, CFA N=6) revealed no significant differences. Exact P-values are provided alongside the raw data in the source data file. Source data are provided as a Source Data file.

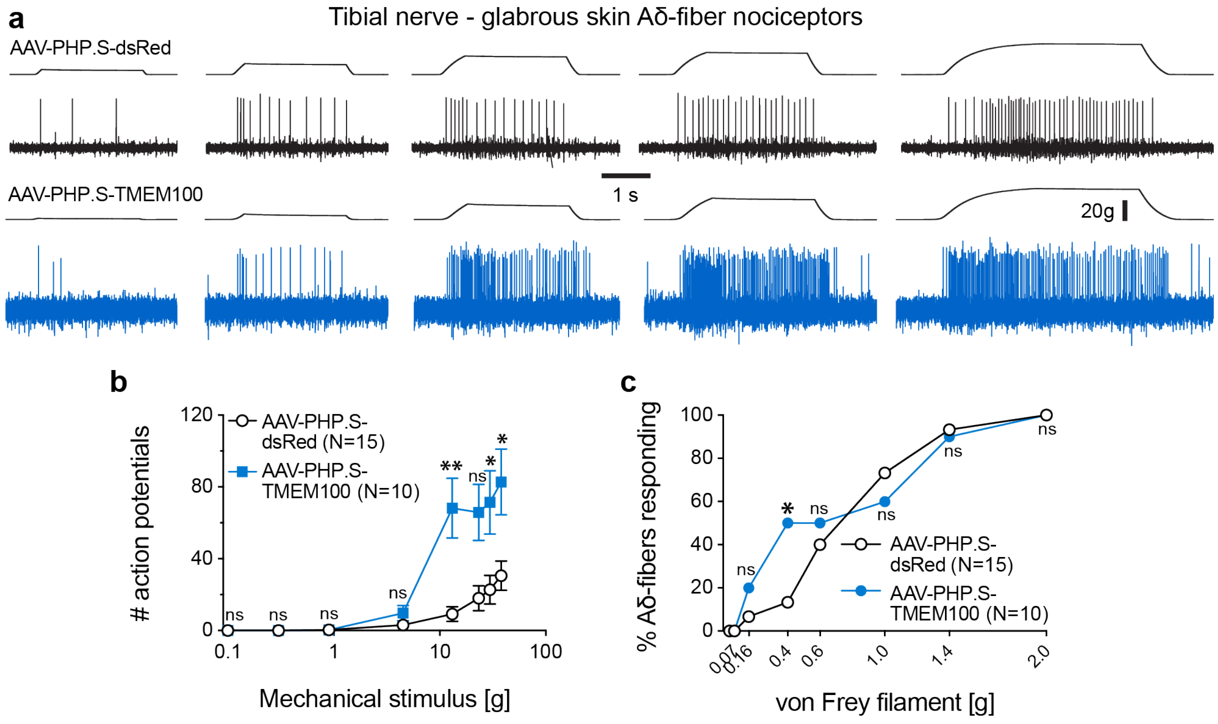

Supplementary Figure. 7: Ex-vivo skin nerve recordings of cutaneous Aδ-fiber nociceptors following AAV-PHP.S-TMEM100-Ires-dsRed knee joint injection. a, Example traces of mechanically-evoked action potentials recorded from single nerve fibers from the tibial nerve of WT mice after knee joint injection of AAV-PHP.S-dsRed control virus (top) or AAV-PHP.S-TMEM100-Ires-dsRed (bottom). **b**, Comparison of the firing rates evoked by a series of ramp-and-hold stimuli with increasing amplitudes that exerted the indicated force to the receptive fields. Symbols represent the mean ± SEM numbers of action potentials, which were compared using multiple two-sided Mann-Whitney tests. P-values from left to right: 0.99, 0.99, 0.61, 0.17, 0.003, 0.067, 0.014, 0.023. Abbreviated P-values (ns, P>0.05; *, P<0.05) and N-numbers are provided in the graph. **c**, Comparison of the proportions of Aδ-fiber nociceptors that respond to mechanical stimulation with the indicated von Frey filaments. The proportions were compared pairwise using the two-sided Chi-square test. P-values from left to right: 0.31, 0.045, 0.62, 0.48, 0.76. Additional information about P-values (ns, P>0.05; *, P<0.05) and N-numbers are provided in the graph. Source data are provided as a Source Data file.
