# Supplementary material for: Role of TMEM100 in mechanically insensitive nociceptor un-silencing
Source: Nat Commun. 2023 Apr 5;14:1899. doi: 10.1038/s41467-023-37602-w (PMC10076432; doi:10.1038/s41467-023-37602-w)
Supplement: Supplementary file 3 — Description of Additional Supplementary Files [file 41467_2023_37602_MOESM3_ESM.pdf]

Description of Additional Supplementary Files

File Name: Supplementary Movie 1
Description: The movie shows an example Catwalk XT analysis of a wildtype mouse three days post intra-articular saline injection. **Top**, video of the mouse walking along the catwalk. Note footprints are detected by the Catwalk XT system and are depicted in green. **Bottom left,** visualization of the spatial and temporal changes in footprint intensity. **Bottom right**, time course of maximum (red line) and mean (shaded areas) of the footprint intensity of the four paws (RF = right forepaw, RH = right hind paw, LF = left forepaw, LH = left hind paw).

File Name: Supplementary Movie 2
Description: The movie shows an example Catwalk XT analysis of a wildtype mouse three days post intra-articular CFA injection. **Top**, video of the mouse walking along the catwalk. Note footprints are detected by the Catwalk XT system and are depicted in green. **Bottom left,** visualization of the spatial and temporal changes in footprint intensity. **Bottom right**, time course of maximum (red line) and mean (shaded areas) of the footprint intensity of the four paws (RF = right forepaw, RH = right hind paw, LF = left forepaw, LH = left hind paw).

File Name: Supplementary Movie 3
Description: The movie shows an example Catwalk XT analysis of a TMEM100KO mouse three days post intra-articular CFA injection. **Top**, video of the mouse walking along the catwalk. Note footprints are detected by the Catwalk XT system and are depicted in green. **Bottom left,** visualization of the spatial and temporal changes in footprint intensity. **Bottom right**, time course of maximum (red line) and mean (shaded areas) of the footprint intensity of the four paws (RF = right forepaw, RH = right hind paw, LF = left forepaw, LH = left hind paw).
