# Supplementary material for: Role of TMEM100 in mechanically insensitive nociceptor un-silencing
Source: Nat Commun. 2023 Apr 5;14:1899. doi: 10.1038/s41467-023-37602-w (PMC10076432; doi:10.1038/s41467-023-37602-w)
Supplement: Supplementary file 7 — Reporting Summary [file 41467_2023_37602_MOESM7_ESM.pdf]

nature portfolio

Corresponding author(s): Stefan G. Lechner

Last updated by author(s): Mar 13, 2023

Reporting Summary

Nature Portfolio wishes to improve the reproducibility of the work that we publish. This form provides structure for consistency and transparency in reporting. For further information on Nature Portfolio policies, see our Editorial Policies and the Editorial Policy Checklist.

Statistics

For all statistical analyses, confirm that the following items are present in the figure legend, table legend, main text, or Methods section.

n/a	Confirmed	
☐	☒	The exact sample size (*n*) for each experimental group/condition, given as a discrete number and unit of measurement
☐	☒	A statement on whether measurements were taken from distinct samples or whether the same sample was measured repeatedly
☐	☒	The statistical test(s) used AND whether they are one- or two-sided<br>*Only common tests should be described solely by name; describe more complex techniques in the Methods section.*
☒	☐	A description of all covariates tested
☐	☒	A description of any assumptions or corrections, such as tests of normality and adjustment for multiple comparisons
☐	☒	A full description of the statistical parameters including central tendency (e.g. means) or other basic estimates (e.g. regression coefficient) AND variation (e.g. standard deviation) or associated estimates of uncertainty (e.g. confidence intervals)
☐	☒	For null hypothesis testing, the test statistic (e.g. *F*, *t*, *r*) with confidence intervals, effect sizes, degrees of freedom and *P* value noted<br>*Give P values as exact values whenever suitable.*
☒	☐	For Bayesian analysis, information on the choice of priors and Markov chain Monte Carlo settings
☒	☐	For hierarchical and complex designs, identification of the appropriate level for tests and full reporting of outcomes
☒	☐	Estimates of effect sizes (e.g. Cohen's *d*, Pearson's *r*), indicating how they were calculated

Our web collection on statistics for biologists contains articles on many of the points above.

Software and code

Policy information about availability of computer code

Data collection	Gait analysis: CatWalkXT10.6<br>Patch-Clamp: Patchmaster (V2x91, HEKA)<br>Skin-Nerve: Labchart 7.2 (AD instruments)<br>Immunofluorescence: LAS X 3.5 (Leica Microsystems)<br>Calcium imaging: ZEN 2 pro software (Carl Zeiss Microscopy GmbH)<br>LABORAS v2.6 (Metris, NL)
Data analysis	Gait analysis: CatWalkXT10.6<br>Patch-Clamp: Fitmaster (V2x91, HEKA)<br>Skin-Nerve: Labchart 7.2 (AD instruments)<br>Immunofluorescence: LAS X 3.5 (Leica Microsystems)<br>Calcium imaging: ZEN 2 pro software (Carl Zeiss Microscopy GmbH)<br>Excel (V16.16.27; Microsoft)<br>Prism Graphpad v8 & v9 (GraphPad Software)<br>Fiji/ImageJ (v2.3.0/1.53f; NIH)<br>LABORAS v2.6 (Metris, NL)

For manuscripts utilizing custom algorithms or software that are central to the research but not yet described in published literature, software must be made available to editors and reviewers. We strongly encourage code deposition in a community repository (e.g. GitHub). See the Nature Portfolio guidelines for submitting code & software for further information.

Data

Policy information about availability of data

All manuscripts must include a data availability statement. This statement should provide the following information, where applicable:

- Accession codes, unique identifiers, or web links for publicly available datasets
- A description of any restrictions on data availability
- For clinical datasets or third party data, please ensure that the statement adheres to our policy

All data supporting the findings of this study are available within the article and its supplementary information files. The RNA sequencing dataset generated in this study are deposited in the Gene Expression Omnibus under accession number GSE199580 [https://www.ncbi.nlm.nih.gov/gds/?term=GSE199580]. Source data are provided with this paper.

Human research participants

Policy information about studies involving human research participants and Sex and Gender in Research.

Reporting on sex and gender	n/a
Population characteristics	n/a
Recruitment	n/a
Ethics oversight	n/a

Note that full information on the approval of the study protocol must also be provided in the manuscript.

Field-specific reporting

Please select the one below that is the best fit for your research. If you are not sure, read the appropriate sections before making your selection.

☒ Life sciences ☐ Behavioural & social sciences ☐ Ecological, evolutionary & environmental sciences

For a reference copy of the document with all sections, see nature.com/documents/nr-reporting-summary-flat.pdf

Life sciences study design

All studies must disclose on these points even when the disclosure is negative.

Sample size	Due to the lack of knowledge of the effect size of TMEM100 knock-out and overexpression, sample size was not predetermied based on power analysis. Previous studies from my lab using the same experimental techniques (Arcourt et al. Neuron 2017, Dhandapani et al. Nat Commun 2018, Gangadharan et al. Nature 2022), however, suggested that between 10 and 15 mice per group are enough to obtain meaningful and reproducible results.
Data exclusions	No data were excluded from Immunohistochemistry, RNAseq calcium imaging and qPCR experiments.<br>From patch clamp and skin-nerve experiments, recordings from cells and nerve fibers that were visibly damaged during the application of incrementing mechanical stimuli were excluded. Moreover, patch-clamp recordings in which the leak current increased or the cell closed during the recording were also not considered for the analysis. From the behavioral analysis, mice that exhibited severe health issues in addition to the knee joint inflammation were excluded form the analysis.
Replication	All electrophysiological, Ca2+ Imaging, IHC and qPCR experiments were performed with tissues and cells from at least 3 mice per group. All replication attempts were successful and no replicates were excluded
Randomization	All mice were age-matched and were randomly assigned to different experimental groups.
Blinding	Except for the behavioral experiments, all experimenters were blinded. In the behavioral experiments blinding was not possible because the CFA-induced knee joint inflammation is so prominent that one can distinguish saline and CFA-treated animals at first glance.

Reporting for specific materials, systems and methods

We require information from authors about some types of materials, experimental systems and methods used in many studies. Here, indicate whether each material, system or method listed is relevant to your study. If you are not sure if a list item applies to your research, read the appropriate section before selecting a response.

Materials & experimental systems

n/a	Involved in the study
☐	☒ Antibodies
☐	☒ Eukaryotic cell lines
☒	☐ Palaeontology and archaeology
☐	☒ Animals and other organisms
☒	☐ Clinical data
☒	☐ Dual use research of concern

Methods

n/a	Involved in the study
☒	☐ ChIP-seq
☒	☐ Flow cytometry
☒	☐ MRI-based neuroimaging

Antibodies

Antibodies used	rat anti-GFP (Nacalai tesque, #04404-84, RRID:AB_10013361<br>rabbit anti-CGRP (ImmunoStar,#24112, RRID:AB_572217)<br>rabbit anti-dsRed (Takara, RRID:AB_2801258)<br>Alexa Fluor 488 conjugated donkey anti-Rat IgG (Thermo Fisher Scientific, #A48269)<br>Alexa 594 conjugated donkey anti-Rabbit IgG (Thermo Fisher Scientific, #A32754)
Validation	anti-GFP: for a list of citations in which this antibody was used and validated please see (https://antibodyregistry.org/search.php?q=AB_10013361)<br>anti-CGRP: The specificity of the antiserum was evaluated by soluble pre-adsorption with the peptides in question at a final concentration of 10-5M. CGRP immunolabeling was completely abolished by pre-adsorption with rat α -CGRP and partially eliminated by pre-adsorption with rat (see https://antibodyregistry.org/search.php?q=AB_572217)<br>anti-dsRed: validated by manufacturer (see https://www.takarabio.com/search-results?term=632392,&tab=product)

Eukaryotic cell lines

Policy information about cell lines and Sex and Gender in Research

Cell line source(s)	HEK293 cells ATCC (#CRL-1573)<br>AAV-293 cells (Agilent, 240073)
Authentication	None of the cell lines used were authenticated.
Mycoplasma contamination	All cell lines were tested negatively for mycoplasma contamination
Commonly misidentified lines<br>(See ICLAC register)	No commonly misidentified cell line was used in this study.

Animals and other research organisms

Policy information about studies involving animals; ARRIVE guidelines recommended for reporting animal research, and Sex and Gender in Research

Laboratory animals	CHRNA3-EGFP mice (Tg(Chrna3-EGFP)BZ135Gsat/Mmnc)<br>8-15 weeks<br><br>CHRNA3-EGFP mice (Tg(Chrna3-EGFP)BZ135Gsat/Mmnc) x TMEM100(B6.Tmem100tm1.1Yjl) x C57BL/6-Tg(SCN10A-Cre)1Rkun/Uhg<br>8-15 weeks<br><br>Tg(Npy2r-cre)SM19Gsat/Mmucd x B6;129S-Gt(ROSA)26Sortm32(CAG-COP4*H134R/EYFP)Hze/J<br>8-15 weeks
Wild animals	No wild animals were used in this study
Reporting on sex	Male and female mice were examined separately and gave identical results.
Field-collected samples	No field collected samples were used in this study.
Ethics oversight	The study was approved by the Regierungspräsidium Karlsruhe under the approval number G16/20

Note that full information on the approval of the study protocol must also be provided in the manuscript.
